# Visual pathway function in adults born preterm with very low birth weight: a two-country birth cohort study

**DOI:** 10.1007/s10633-025-10025-2

**Published:** 2025-05-28

**Authors:** Anna P. M. Jørgensen, Maarit Kulmala, Dordi Austeng, Trond Sand, Arnstein Grøtting, Kari Anne I. Evensen, Eero Kajantie, Anna Majander, Tora Sund Morken

**Affiliations:** 1 Department of Neuromedicine and Movement Science, Norwegian University of Science and Technology (NTNU), 7491 Trondheim, Norway; 2 Welfare Epidemiology and Monitoring Unit, Finnish Institute for Health and Welfare, Helsinki, Finland; 3 Department of Ophthalmology, Helsinki University Hospital, Helsinki, Finland; 4 Department of Ophthalmology, St. Olavs Hospital, Trondheim University Hospital, Trondheim, Norway; 5 Department of Neurology and Clinical Neurophysiology, St. Olavs Hospital, Trondheim University Hospital, Trondheim, Norway; 6 Department of Clinical and Molecular Medicine, NTNU, Trondheim, Norway; 7 Children’s Clinic, St. Olavs Hospital, Trondheim University Hospital, Trondheim, Norway; 8 Department of Rehabilitation Science and Health Technology, Oslo Metropolitan University, Oslo, Norway; 9 Clinical Medicine Research Unit, MRC Oulu, Oulu University Hospital and University of Oulu, Oulu, Finland; 10 Children’s Hospital, Helsinki University Hospital, Helsinki, Finland; 11 University of Helsinki, Helsinki, Finland

**Keywords:** Preterm, Very low birth weight, Adult, Pattern reversal electroretinogram, Pattern reversal visual evoked potential

## Abstract

**Purpose:**

To investigate if preterm birth with very low birth weight (VLBW; birth weight < 1500 g) affects macular and visual pathway function in an adult population and explore if best corrected visual acuity (BCVA) was associated with any of the electrophysiologic responses.

**Methods:**

Fifty participants born preterm with VLBW and 77 term-born controls years were recruited when aged 31-41 years from the Helsinki Study of Very Low Birth Weight Adults (Finland) and the NTNU Low Birth Weight Life study (Norway) studies. Pattern reversal electroretinogram (PR-ERG), visual evoked potential (PR-VEP) and BCVA were examined. PR-ERG components (P50, N95 peak time, amplitude and N95:P50 amplitude ratio) and PR-VEP components (N75, P100 and N145 peak time and amplitude) in the better-seeing eye were compared between the groups, and association with BCVA was examined.

**Results:**

The VLBW group showed longer N145 peak time compared to the control group (mean difference 6.8 ms, CI 2.0 to 11.5, p = 0.006) and lower N95:P50 amplitude ratio (CI − 0.3 to − 0.1, p = 0.003). Otherwise, both groups showed similar electrophysiological waveforms. No relationship was found between electrophysiologic responses and BCVA. BCVA was normal in birth groups and showed no group difference.

**Conclusion:**

The responses in the primary visual cortex (N75 and P100) to visual stimuli presented to the better-seeing eye were similar in adults born preterm with VLBW and term-born controls. However, in the VLBW group, there was an indication that subtle electrophysiological deviation may exist at a higher cortical level (N145) and in the ganglion cell response in the macula. These significant differences were not related to reduced visual acuity.

**Supplementary Information:**

The online version contains supplementary material available at 10.1007/s10633-025-10025-2.

## Introduction

In recent decades, there has been increasing recognition that children born preterm are at greater risk of long-term neurodevelopmental sequelae than those born at term [1]. Preterm birth alters the growth environment of the child and interrupts developmental processes, including neuronal differentiation and cell migration in all neural tissues [2]. The visual pathway from the retina to the primary visual cortex undergoes significant development during the prenatal and neonatal period and is particularly vulnerable [3–5]. Functional changes in the retina and post-retinal pathway can be detected using visual electrophysiology, such as electroretinogram (ERG) and visual evoked potential (VEP). Both measures mainly reflect the functional state of the so-called parvocellular system and ventral stream [6], which is specific to central vision function as well as color and form perception [7, 8]. Most changes in visual pathway maturation are seen during early childhood with decreasing ERG and VEP peak time and increasing amplitude, but further electrophysiologic maturation proceeds slowly until adulthood [6, 9].

Adolescents born preterm have been shown to have altered ERG (full-field and multifocal), suggesting discrete retinal, mostly macular, dysfunction [10–12]. Furthermore, previous studies that have explored VEP responses have found changed cortical responsiveness in otherwise healthy (absence of retinal and cerebral abnormalities) children born preterm [13], longer peak times [14] and reduced amplitude in adolescents born preterm tested with pattern reversal VEP (PR-VEP) [15]. In contrast to these findings, normal full-field ERG [15] and high-contrast VEP [16] have also been reported in children born preterm. Interestingly, several studies have reported that the extrauterine life appears to accelerate the maturation of the visual system in children born preterm and that they by term-equivalent age reach the same degree of maturation found in children born at term, indicating a catch-up effect [16–19]. Although these results suggest that children born preterm may overcome visual development delay with time, it seems that the immaturity of the visual pathway does not completely disappear.

The functional state of the visual pathway in adults born preterm has not been assessed by other previous studies in mid-adulthood. To this end, the current study sought to assess if preterm birth affects the pattern reversal ERG (PR-ERG) and PR-VEP activity in adults born preterm with very low birth weight compared to controls born at term at 31–41 years. The main objectives were to assess group differences in PR-ERG P50 and N95 amplitudes and in the main PR-VEP variables P100 peak time and amplitude. Secondary objectives were to estimate group differences for PR-ERG P50, N95 peak time, N95:P50 amplitude ratio and PR-VEP N75 peak time and N145 peak time and amplitude. Lastly, this study also aimed to explore if best corrected visual acuity was associated with any of the electrophysiologic responses.

## Method

### Study design

This study is part of a follow-up assessment of two longitudinal birth cohort studies [20], the Helsinki Study of Very Low Birth Weight Adults (HeSVA) in Finland [21] and the NTNU Low Birth Weight in a Lifetime Perspective study (NTNU LBW Life) in Norway [1]. The HeSVA is a geographically defined birth cohort, while the NTNU LBW Life is a hospital-defined birth cohort of VLBW (birthweight < 1500 g) participants and a geographically defined cohort of controls born at term. All examinations were performed as a joint data collection with harmonized study protocols and methods between 11 September 2019 to 5 July 2021. Examiners were masked for group status.

### Study participants

**Fig. 1:**
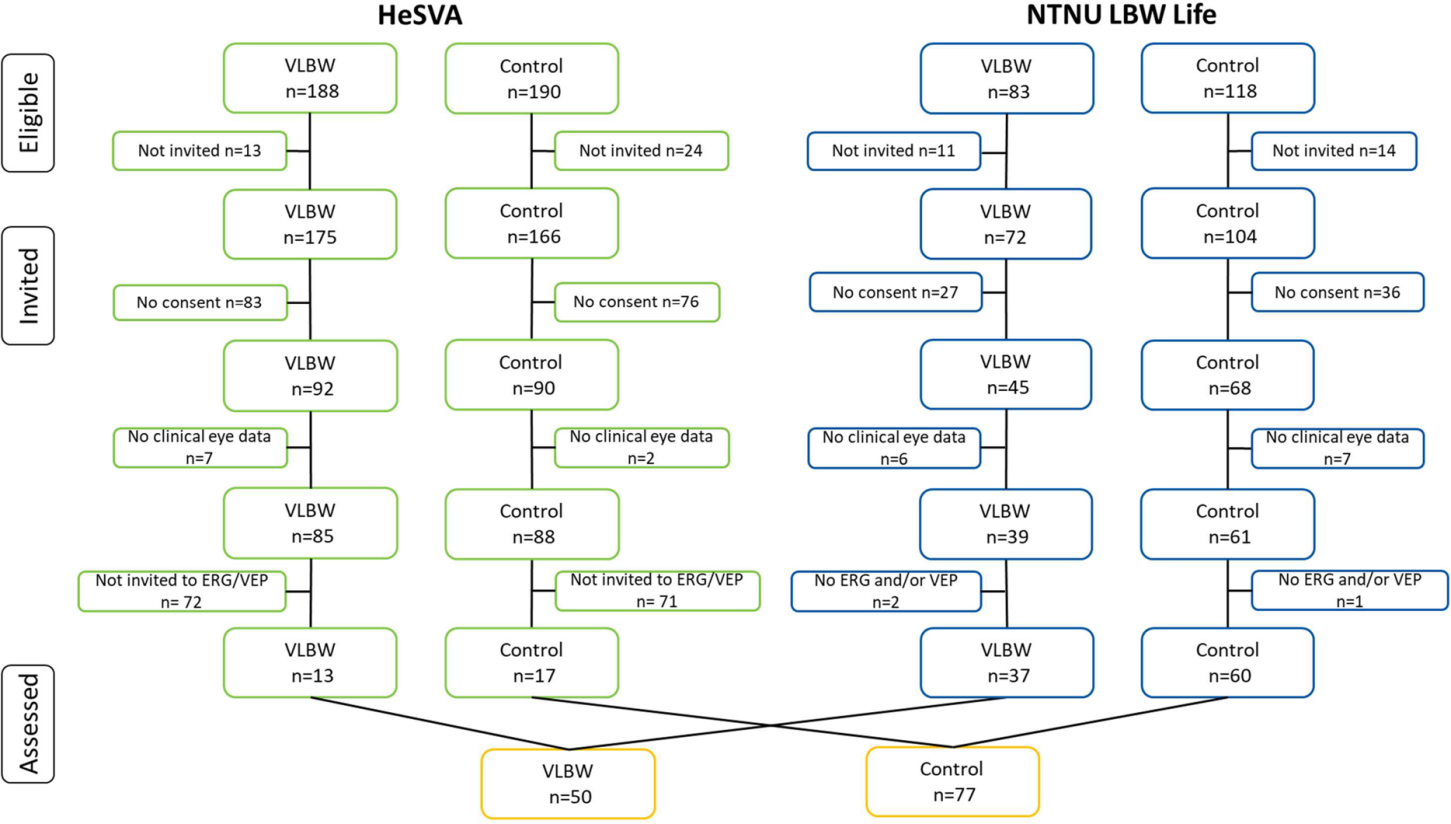
Flow diagram of adults born VLBW and term-born control participants. *HeSVA* Helsinki Study of Very Low Birth Weight Adults; *NTNU LBW Life* NTNU Low Birth Weight in a Lifetime Perspective study; *ERG* electroretinogram; *VEP* visual evoked potential

The participants were recruited from the HeSVA and NTNU LBW Life. The HeSVA included children born between 1978 to 1985 and the NTNU LBW Life included children born between 1986 to 1988. The clinical ophthalmological examination involved 124 participants born preterm with VLBW and 149 control participants born at term. Results of best corrected visual acuity (BCVA) have been published [22]. Of the 173 HeSVA participants, 30 participants (13 VLBW and 17 controls) were selected at random and invited to electrophysiological examination. VLBW participants were randomly selected among those born with a GA < 29 weeks. All 100 participants (39 VLBW and 61 Control) from NTNU LBW Life were invited to this examination, where two VLBW and one control participant did not consent. In total, 50 VLBW participants and 77 control participants had PR-ERG, PR-VEP and BCVA data. Non-participants were defined as those who did not have the electrophysiological examination, including 143 HeSVA participants who were not invited and three NTNU LBW Life participants who did not consent to this examination. Figure 1 presents a detailed flow of participants.

### Background data

Background data had previously been collected from medical records in both study cohorts. The following characteristics were obtained: Birth weight, gestational age at birth and sex. Additionally, characteristics included self-reported previous trauma, ocular surgery, diagnosed multiple sclerosis (MS), previous optic neuritis and presence of neurosensory impairments (NSI). NSI was defined as blindness, use of a hearing aid, diagnosed cerebral palsy, and/or IQ score below 70 defined by self-report in young adulthood (HeSVA) or clinically assessed 19, 14, or 5 years of age (NTNU LBW Life). At the time the participants were born, screening programs for retinopathy of prematurity (ROP) were not yet in effect in Finland and Norway, and data on ROP were not available in medical records to an extent that allowed analyses. None of the participants had been treated for ROP. The intraocular structures, i.e. the anterior and posterior segment, were assessed by slit lamp ophthalmoscopy where none of the participants had any pathological findings in the anterior or posterior segment [22].

### Best corrected visual acuity

Best corrected visual acuity was assessed according to the Early Treatment Diabetic Retinopathy Study (ETDRS) protocol [23]. ETDRS letter score values were used to estimate BCVA. The eye with the better ETDRS letter score was chosen for further analyses. If the ETDRS letter score was equal in both eyes, the right eye was chosen as better eye.

### Electrophysiologic measurements

During testing, participants wore optimal refraction with an additional +1 diopter spherical correction corresponding to 1 m testing distance. PR-ERG and PR-VEP were assessed according to the International Society for Clinical Electrophysiology of Vision (ISCEV) standards [24, 25] except for the PR-VEP checkwidth (see below). In HeSVA, a RETI-port/scan 21 workstation (Roland Consult Electrophysiology and Imaging, Brandenburg an der Havel, Germany) with IIYAMA ProLite B198OSD, Camera (LA-S, SN53-99-7.5-199004, Germany) screen as the pattern generator. In the NTNU LBW Life study, a Dantec™ Keypoint workstation (v2.32, San Carlos, CA, USA) with a View Sonic Graphics Series G70fmb cathode-ray tube screen as the pattern generator was used. Electrodes were placed according to the 10–20 system on occipital, frontal, and parietal areas (Oz, Fz and Pz) [26]. Both tests were recorded from the occipital (Oz) midline and referred to the mid-frontal electrode (Fz). The parietal to mid-frontal recording (Pz-Fz) was also available at NTNU to aid the identification of P100 component peak at Oz. Before performing PR-ERG, corneal electrodes (DTL plus electrode, Diagnosys LLC, 55 Technology Drive, Lowell, MA 01851, USA) were placed in the inferior fornix and ipsilateral temporal reference electrodes were placed on the skin on both sides. Both eyes were tested at the same time when recording PR-ERG and one eye at a time, right eyes first, when performing PR-VEP. A 1 Hz–1 kHz filter was used and the electrode impedance was less than 10 kΩ (≤ 5 kΩ in the NTNU LBW life study). Testing was performed in dim ambient illumination with photopic luminance in the white areas of the checkerboard pattern > 80 cd/m^2^, contrast > 80% and 18° × 14° field size.

Participants were placed 1 m from the screen in a relaxed position and instructed to fixate on a red target at the center of the checkerboard pattern display. When recording PR-ERG the size of the check pattern was 48′ in HeSVA and 60′ in the NTNU LBW Life study. When recording PR-VEP the size of the check pattern used in HeSVA was 60′ (ISCEV Standard large checks) and 33′ in the NTNU LBW Life study. These check pattern sizes were considered to be comparable regarding expected peak time and amplitude parameters [27, 28]. Medium-size checks around 0.5° have been used for decades as our internal standard at NTNU to avoid problems with visual acuity for small checks and partial parafoveal stimulation for large checks, as previously recommended [29]. The pattern reversal during recording was 4 per second (4.0 rps) with 200 stimulations per run for PR-ERG and 1.5 rps with 160 stimulations per run for PR-VEP. At least two runs for each eye were recorded to assess reproducibility and two equal responses were required for the recording to be considered reliable for analyses. Representative PR-ERG and PR-VEP waveforms are presented in Fig. 1S. Data were evaluated by an experienced clinical electrophysiologist at each site, blinded to group, who identified the recorded components; P50 and N95 from PR-ERG and N75, P100, and N145 peaks from PR-VEP. All curves were later scrutinized by another blinded senior clinical neurophysiologist for final peak identification. Two or three reliable response runs were then averaged by the software to get a final curve for the components. The time from stimulus onset to maximum amplitude (peak time) was calculated by the software as well as the amplitude of the components. Amplitudes were calculated from peak-to-peak, e.g. the P50 amplitude was calculated from the preceding peak, the N35 amplitude, to the peak of P50 (the N35-P50 amplitude) and the N95 amplitude was calculated from the peak of P50 to the peak of N95 (the P50-N95 amplitude). In two cases, P100 peak times were calculated manually with the use of the Chiappa method, with flank extrapolation in cases where a so-called “W” or bifid pattern was observed [27]. The peak time (ms) and peak-to-peak amplitude (μV) of the components were obtained and data from the better eye were used for analyses. The scorers were blinded to group status. Because amplitude distributions are skewed, we used the square root of the N95 and P50 amplitude values to calculate the (transformed) N95:P50 amplitude ratio for statistical analysis.

### Statistical analyses

Statistical analyses were performed with SPSS software 28.0 (IBM Statistics, NY, USA) and RStudio 4.1.2 (PBC, MA, USA). Two-sided p-values < 0.05 were considered statistically significant. In addition, adjustments for multiple comparisons were performed and Benjamin–Hochberg adjusted p-values are presented. Normality was assessed by visual inspection of Q–Q plots of standardized residuals. In the cases where deviations from normality were found, we applied bootstrapping with B = 2000 samples and bias-corrected and accelerated (Bca) confidence intervals are reported. General linear models were used to assess the mean difference between the groups (VLBW vs control) for PR-ERG and PR-VEP responses and BCVA. Linear regression analyses were used to explore the relationship between BCVA and PR-ERG and PR-VEP peak times and amplitudes, which were entered as dependent variables (one at a time). To investigate possible VEP-ERG dependence, the association between the significant VEP and ERG responses was investigated by linear regression. We adjusted for age, sex, and cohort in all analyses, and we performed sensitivity analyses where we excluded participants with previous ocular surgery, diagnosed MS, optic neuritis and NSI. In addition, BCVA in non-participants was compared with BCVA in participants.

### Ethics

The study was approved by the local ethics committees at each site; the Ethics Committee IV of Helsinki University and Uusimaa Hospital District (HUS/1157) in Finland and the Regional Committees for Medical and Health Research Ethics in Central Norway (REK/23879). The study complied with the guidelines of the Declaration of Helsinki. The study had institutional approval by Helsinki University Hospital, Helsinki (Finland) and St. Olavs hospital, Trondheim University Hospital, Trondheim (Norway). Informed written consent was obtained from all participants.

## Results

### Participant characteristics

Background characteristics of the VLBW and the control group are presented in Table 1. There was a slightly larger proportion of females among participants. Age at examination was similar between the groups. None of the participants reported having previous ocular trauma. Five participants (VLBW n = 4, control n = 1) had previous refractive surgery, two VLBW participants had NSI, one had diagnosed MS and one had previous optic neuritis.

**Table 1:** Background characteristics of adults born preterm with very low birth weight and controls born at term

	VLBW (n = 50)		Control (n = 77)	
	Mean (SD)		Mean (SD)	
Birth weight (g)	1210	(212)	3664	(460)
Gestational age (weeks)	29.2	(2.5)	39.9	(1.2)
Age at follow-up (years)	33.9	(2.8)	33.9	(2.8)

	n	(%)	n	(%)
Sex (female)	32	(64)	40	(51)
Trauma (yes)	0	(0)	0	(0)
Ocular surgery (yes)	4	(0.08)	1	(0.01)
NSI (yes)	2	(4)	0	(0)
MS (yes)	1	(2)	0	(0)
Optic neuritis (yes)	1	(2)	0	(0)
HeSVA (yes)	13	(26)	17	(22)
NTNU LBW Life (yes)	37	(74)	60	(78)

*GA* gestational age; *SD* standard deviation; *NSI* neurosensory impairments; *MS* multiple sclerosis; *HeSVA* Helsinki Study of Very Low Birth Weight Adults; *NTNU LBW Life* NTNU Low Birth Weight in a Lifetime Perspective study

### Best corrected visual acuity

Mean BCVA letter score in the better eye was similar between the groups with a mean difference of 1.1 (95% CI − 2.7 to 0.4) (Table 2).

**Table 2:** Electrophysiologic and  visual acuity data and group differences for the better eyes of  adults born preterm with VLBW and controls born at term

	VLBW (n = 50)		Controls (n = 77)					
	Mean	(SD)	Mean	(SD)	Mean difference	95% CI	p-value	p-value ^adj^
Visual function								
BCVA score*	87.8	(4.9)	88.7	(4.2)	− 1.1	(− 2.7 to 0.4)	0.165	0.303
PR-ERG^a^								
P50 peak time (ms)	51.6	(3.2)	51.3	(2.9)	0.3	(− 0.7 to 1.4)	0.513	0.733
N95 peak time (ms)	89.6	(5.6)	89.1	(5.0)	0.6	(− 1.4 to 2.5)	0.570	0.733
P50 amplitude (µV)	4.0	(1.5)	3.8	(1.3)	0.1	(− 0.4 to 0.5)	0.740	0.740
N95 amplitude (µV)	4.9	(2.6)	5.3	(1.9)	− 0.7	(− 1.4 to 0.1)	0.068	0.249
sqr N95:P50 amplitude ratio^b^	1.05	(0.34)	1.21	(0.24)	− 0.2	(− 0.3 to − 0.1)	**0.003**	**0.033**
PR-VEP^c^								
N75 peak time (ms)	70.9	(5.4)	71.6	(4.4)	− 0.4	(− 2.0 to 1.3)	0.666	0.733
P100 peak time (ms)	101.6	(5.6)	100.3	(5.3)	1.5	(− 0.3 to 3.3)	0.101	0.268
N145 peak time (ms)	149.0	(15.7)	142.1	(13.3)	6.8	(2.0 to 11.5)	**0.006**	**0.033**
P100 amplitude (µV)	10.0	(5.2)	9.9	(4.8)	0.4	(− 1.2 to 2.0)	0.621	0.733
N145 amplitude (µV)	11.5	(5.2)	12.4	(5.4)	− 1.4	(− 3.3 to 0.4)	0.122	0.268

Mean difference analysed between the two groups (VLBW vs control) with a general linear model with applied bootstrapping adjusting for age, sex and cohort

*BCVA* best corrected visual acuity; *VLBW* very low birth weight; *SD* standard deviation; *CI* confidence interval; *p-value *^*adj*^ Benjamin Hochberg adjusted p-values; *PR-ERG* pattern reversal electroretinogram; *PR-VEP* pattern reversal visual evoked potential; *sqr* square root; *ms* milliseconds; *µV* microvolt

*ETDRS letter score

^a^Missing data for two VLBW and one control participant

^b^The amplitude ratio is calculated as the N95 amplitude divided by the P50 amplitude of square root transformed values

^c^Missing data for one control participant

Two-sided p-values < 0.05 were considered statistically significant

### Electrophysiological responses

The N95:P50 amplitude ratio was lower in the VLBW group compared to the control group, with a mean difference of − 0.2 (95% CI − 0.3 to − 0.1, p = 0.003). In addition, the VLBW group had longer PR-VEP N145 peak time compared with the control group with a mean difference of 6.8 ms (95% CI 2.0 to 11.5, p = 0.006). Other peak times and amplitudes were similar between groups. After adjusting for multiple comparisons, adjusted p-values indicated similar significant differences in the electrophysiological responses (Table 2).

### Associations between electrophysiological responses and visual acuity

**Fig. 2:**
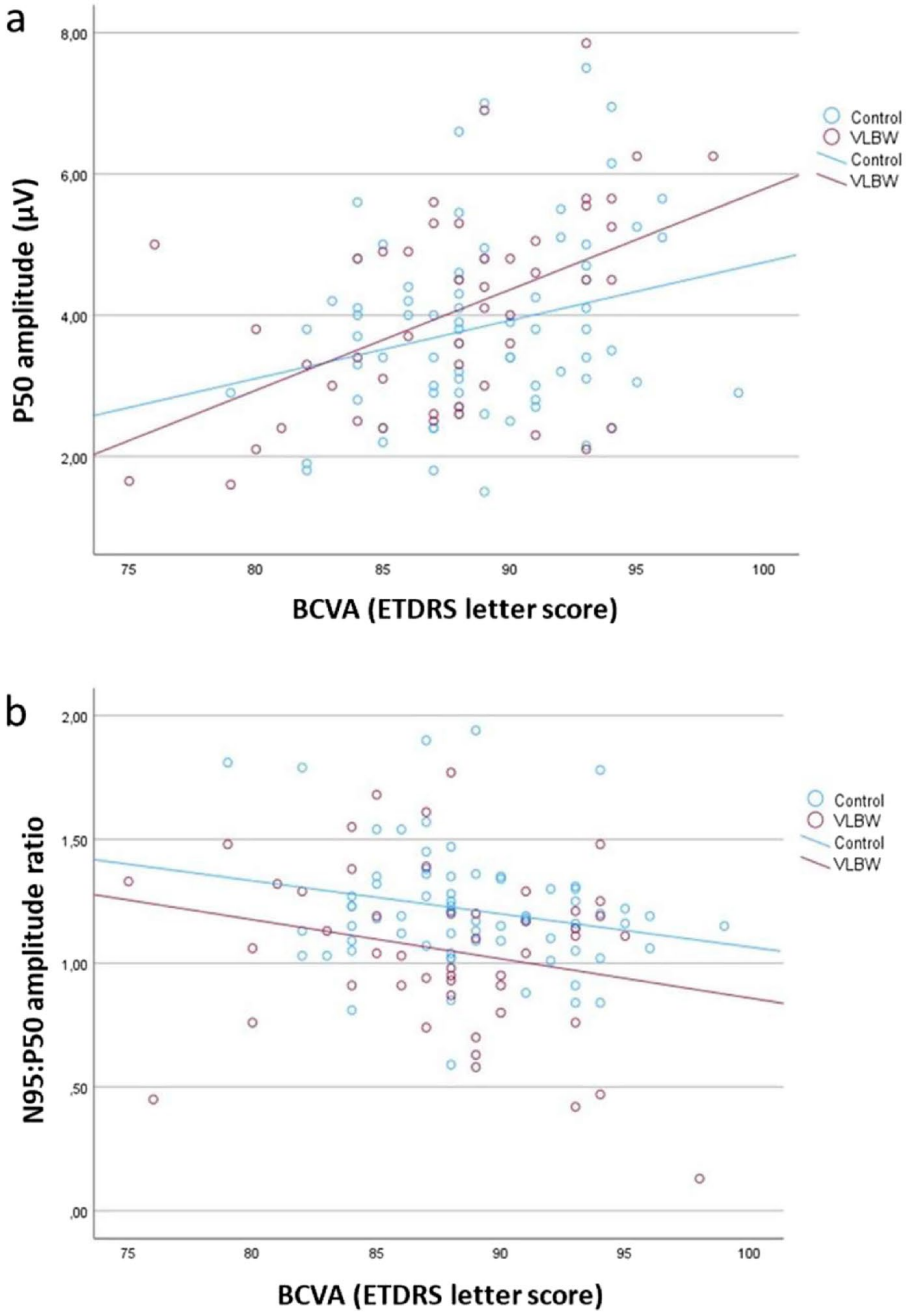
**a** Relationships for the better eye between the P50 amplitude and BCVA, and **b** relationship between the square root transformed N95:P50 amplitude ratio and BCVA in the VLBW and control group. *BCVA* best corrected visual acuity; *ETDRS* Early Treatment Diabetic Retinopathy Study; *VLBW* very low birth weight; *µV* microvolt

A higher P50 amplitude and a lower N95:P50 amplitude ratio were associated with a better BCVA letter score in the VLBW group. A significant association was also found between the ratio and BCVA in controls. We did not find any association between PR-VEP responses and BCVA. After adjusting for multiple comparisons, adjusted p-values indicated that there were not any significant association between electrophysiologic responses and BCVA (Table 3). Figure 2 illustrates the relationship between the P50 amplitude and BCVA (Fig. 2A), as well as the relationship between the N95:P50 amplitude ratio and BCVA (Fig. 2B).

**Table 3:** Linear regression coefficients for the association between electrophysiological response measures and visual outcome

Electrophysiologic response	Visual outcome	VLBW (n = 50)				Controls (n = 77)			
		B	(95% CI)	p-value	p-value^adj^	B	(95% CI)	p-value	p-value^adj^
PR-ERG^a^									
P50 peak time (ms)	BCVA score	− 0.03	(− 0.22 to 0.16)	0.761	0.941	− 0.02	(− 0.22 to 0.15)	0.834	0.927
N95 peak time (ms)	BCVA score	0.02	(− 0.38 to 0.45)	0.929	0.941	− 0.10	(− 0.43 to 0.19)	0.514	0.734
P50 amplitude (µV)	BCVA score	0.10	(0.02 to 0.17)	**0.025**	0.145	0.05	(− 0.02 to 0.11)	0.180	0.733
N95 amplitude (µV)	BCVA score	0.11	(− 0.04 to 0.20)	0.083	0.277	− 0.06	(− 0.20 to 0.09)	0.410	0.733
sqr N95:P50 amplitude ratio^b^	BCVA score	− 0.02	(− 0.03 to − 0.003)	**0.029**	0.145	− 0.02	(− 0.03 to − 0.003)	**0.029**	0.290
PR-VEP^c^									
N75 peak time (ms)	BCVA score	− 0.13	(− 0.56 to 0.24)	0.538	0.897	0.04	(− 0.35 to 0.35)	0.976	0.976
P100 peak time (ms)	BCVA score	0.05	(− 0.35 to 0.38)	0.820	0.941	− 0.04	(− 0.33 to 0.32)	0.787	0.927
N145 peak time (ms)	BCVA score	− 0.75	(− 1.94 to 0.31)	0.216	0.540	− 0.29	(− 0.97 to 0.45)	0.440	0.733
P100 amplitude (µV)	BCVA score	− 0.16	(− 0.53 to 0.09)	0.331	0.662	0.15	(− 0.10 to 0.41)	0.251	0.733
N145 amplitude (µV)	BCVA score	0.01	(− 0.35 to 0.25)	0.941	0.941	0.11	(− 0.17 to 0.42)	0.440	0.733

Regression coefficients (B are the estimated slopes per change in EDTRS letter score) and bias-corrected and accelerated (Bca) bootstrap confidence intervals (CI) in a linear regression model with PR-ERG and PR-VEP responses (one at a time) in the better eye, as dependent variable. Group (VLBW vs control) and visual outcome (BCVA) were entered as covariates, adjusted for age, sex and cohort

*BCVA* best corrected visual acuity in the better eye; *VLBW* very low birth weight; *SD* standard deviation; *CI* confidence interval; *p-value *^*adj*^ Benjamin Hochberg adjusted p-values; *PR-ERG* = pattern reversal electroretinogram; *PR-VEP* pattern reversal visual evoked potential; *sqr* square root; *ms* milliseconds; *µV* microvolt

^a^Missing data for two VLBW and one control participant

^b^The amplitude ratio is defined as the N95 amplitude divided by the P50 amplitude of square root transformed values

^c^Missing data for one control participant

Two-sided p-values < 0.05 were considered statistically significant

No relationship was found between the PR-VEP N145 peak time and PR-ERG N95:P50 amplitude ratio (95% CI − 13.43 to 3.33, p = 0.235).

### Sensitivity and non-participant analyses

When we excluded the five participants with previous refractive surgery, the two participants with NSI, diagnosed MS and previous optic neuritis, the results remained the same. Participants in the VLBW group had a better BCVA compared to non-participants with a mean difference in ETDRS letter score of 6.1 (95% CI 0.4 to 11.0), while the BCVA was similar for participants and non-participants in the control group.

## Discussion

This study revealed mainly similar PR-ERGs and PR-VEPs in adults born preterm with VLBW compared with adults born at term and all of the clinically used measures were similar between the groups. However, we found that the VLBW group displayed a 7 ms longer PR-VEP N145 peak time than the control group and had a lower N95:P50 amplitude ratio by 0.2. No relationship was found between electrophysiologic responses and BCVA.

Both P50 and N95 components reflect retinal ganglion cell (RGC) activity and are sensitive indicators of macular function. The N95 component is thought to be generated in relation to the RGC, with perhaps 70% of the P50 component being related to the RGC with the residual response generated by macular photoreceptor cells [30, 31]. The P50 component is affected by disease in the macula, where the changes are usually confined to the amplitude of this component, resulting in a reduced P50 amplitude [32]. This usually occurs together with a reduction in N95, where the relationship between the N95 and P50, i.e. N95:P50 ratio, is not reduced [30]. In contrast, a selective reduction of the N95 amplitude with preservation of the P50 can be observed in cases of primary RGC dysfunction where the N95:P50 is reduced [33]. Both groups had similar peak times for the P50 and N95 components, suggesting that the RGC activation, in response to a visual stimulus, was functioning well with no group difference. Thus, it seems that the function of the macula overall is well preserved in adults born preterm with VLBW and similar to adults born at term.

However, we found a lower N95:P50 amplitude ratio in the VLBW group compared to the control group, mainly due to a change of the N95 amplitude in the VLBW group. This could indicate the presence of subtle abnormality or perhaps simply differences in the ganglion cell signal transduction in the VLBW group. It has been suggested that the spatial locations of ganglion cell axons may have a role in the signal formation [34]. This might explain our findings, as preterm adults have an altered location of the axons in the macula compared to controls born at term, with less space available for synaptic communication [35]. However, no relationship between electrophysiological responses and BCVA was revealed. Thus, premature birth seems to be linked to subtle macular-driven ganglion cell dysfunction, but not necessarily to reduced uniocular visual acuity in the better seeing eye.

Neuroimaging studies in adolescents born preterm have delineated brain asymmetries and aberrant connectivity, suggesting that improvements might be achieved by establishing unusual patterns of connectivity [36] perhaps due to a prolonged period of plasticity [37]. Previous studies have shown improvements in the visual pathway maturation over time, especially in the parvocellular system and ventral stream in children born preterm [16, 38, 39]. The peak time of the PR-VEP components indicates the velocity of the neural transmission in the post-retinal part of the visual pathway. The primary visual cortex (striate cortex) is considered to be the generator for the N75 and the early part of the P100 components, while the N145 component is considered to be a mixture of components generated partly from striate and partly from extrastriate visual cortices [40]. Delayed N145 in migraine has for instance been attributed to the reduction of the early parvocellular “N130 subcomponent” and the subsequent dominance of the later extrastriate magnocellular “N180 subcomponent” of N145 [41]. We found no association between the N95:P50 amplitude ratio and the N145 peak time, suggesting that the observed macula-driven ganglion cell and cortical electrophysiological deviations may represent physiologically independent features.

The N75 and P100 peak times were similar between adults born preterm and at term in our study. This is not surprising in this subsample of the cohorts, because both groups displayed similar BCVA which depends on the integrity of the visual pathway function.

However, in our study we also observed that the N145 component was delayed in the VLBW compared to the control group, suggesting that there may be subtle alterations in the visual postprocessing of the higher-order visual system that persist into adulthood. Previous studies of individuals born preterm have in general focused on components that reflect primary visual cortex activity [15, 42, 43]. The N145, which is affected, reflects higher-order visual processing and is known to be variable. Therefore, it is not often used in clinical settings [44]. Thus, the clinical significance of our finding with a 7 ms group difference between VLBW and controls in N145 peak time remains to be explored.

## Strengths and limitations

Strengths of this study include its prospective design with well-characterized birth cohorts and controls recruited at the same time and geographical location as the VLBW participants. Furthermore, we performed both PR-ERG and PR-VEP, performed blinded component measurements, and included all available components from these tests along with BCVA. Thus, we were able to explore the visual pathway function, both retinal and post-retinal, and explore if the visual pathway function was associated with BCVA outcome. To our knowledge, this has not been done in any prior study of adults born preterm with VLBW. Lastly, we used the better eye to evaluate the best possible function of the visual pathway in VLBW individuals. Thus, our findings do not overestimate the real-life effects of preterm birth on visual pathway function. However, because only the eye with the best BCVA was analyzed, our results may have underestimated the group difference. PR-ERG and PR-VEP were evoked by different pattern sizes in the two cohorts. However it is probable that the components are comparable due to the use of moderate to large patterns (> ~ 30′) that give more robust responses in participan s with normal visual acuity, i.e. BCVA > 0.0 logMAR. Reduced vision, < 0.2 logMAR, is known to affect responses to pattern stimuli [27, 28]. The ophthalmologic assessment was performed by ophthalmologists and electrophysiologists masked for group status. In addition, one senior electrophysiologist performed a final evaluation of all evoked potentials from both cohorts to avoid possible inter-observer bias. On the other hand, attrition bias could have affected our results because VLBW non-participants had poorer BCVA than VLBW participants. This does not weaken our statistically significant findings, but rather the opposite, as we might have underestimated the total group difference. We adjusted p-values for multiple comparisons with the Benjamin–Hochberg method [45]. This method decrease the likelihood of Type I errors, but do so at the expense of increasing the likelihood of Type II errors [46], and results are presented with both unadjusted and Benjamin–Hochberg adjusted p-values.

## Conclusion

Pattern VEP peak time from the primary visual cortex in adults born preterm with VLBW was similar to adults born at term, in groups with similar BCVA. However, the VLBW group may have slightly altered macula-driven ganglion cell function, seemingly not related to visual acuity. A subtle electrophysiological deviation evoked from a higher cortical level was also observed. The observed changes, reflecting minor neurophysiological deviations, are probably of limited clinical significance. Additional research is needed to identify the mechanisms underlying the observed deviations in the visual pathway function, of VLBW individuals born preterm.

## Supplementary Information

Below is the link to the electronic supplementary material.

Supplementary file1 (PNG 463 kb)
**Figure 1S** Examples of mean pattern reversal ERG (PR-ERG) and VEP (PR-VEP) responses from two representative VLBW subjects, one in HeSVA (**a**, **b**) and one in NTNU LBW Life (**c**, **d**). *HeSVA* Helsinki Study of Very Low Birth Weight Adults; *NTNU LBW Life* NTNU Low Birth Weight in a Lifetime Perspective study; *PR-ERG* pattern reversal electroretinogram; *PR-VEP* pattern reversal visual evoked potential; *µV* microvolt; *ms* milliseconds; *D* division.

Supplementary file2 (PNG 391 kb)
**Figure 2S.** Examples of mean pattern reversal ERG (PR-ERG) and VEP (PR-VEP) responses from two representative control subjects, one in HeSVA (**a**, **b**) and one in NTNU LBW Life (**c**, **d**). Abbreviations: *HeSVA* Helsinki Study of Very Low Birth Weight Adults; *NTNU LBW Life* NTNU Low Birth Weight in a Lifetime Perspective study; *PR-ERG* pattern reversal electroretinogram; *PR-VEP* pattern reversal visual evoked potential; *µV* microvolt; *ms* milliseconds; *D* division.
