# Supplementary figures and images for: Visual pathway function in adults born preterm with very low birth weight: a two-country birth cohort study
Source: Doc Ophthalmol. 2025 May 28;151(1):19–30. doi: 10.1007/s10633-025-10025-2 (PMC12334524; doi:10.1007/s10633-025-10025-2)

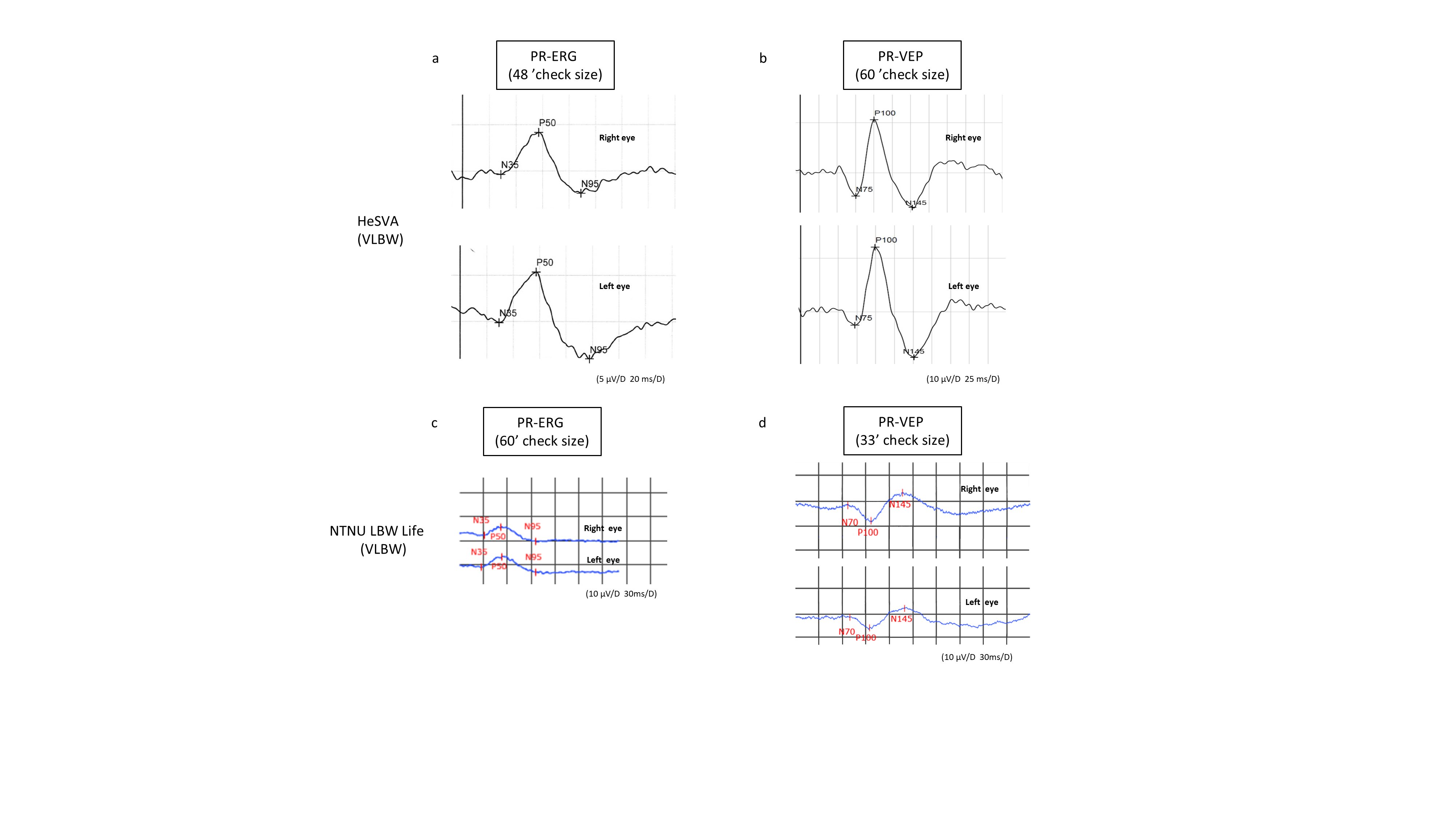

Supplement: Supplementary file 1 — Supplementary file1 (PNG 463 kb) Figure 1S Examples of mean pattern reversal ERG (PR-ERG) and VEP (PR-VEP) responses from two representative VLBW subjects, one in HeSVA (a, b) and one in NTNU LBW Life (c, d). HeSVA Helsinki Study of Very Low Birth Weight Adults; NTNU LBW Life NTNU Low Birth Weight in a Lifetime Perspective study; PR-ERG pattern reversal electroretinogram; PR-VEP pattern reversal visual evoked potential; µV microvolt; ms milliseconds; D division. [file 10633_2025_10025_MOESM1_ESM.png]

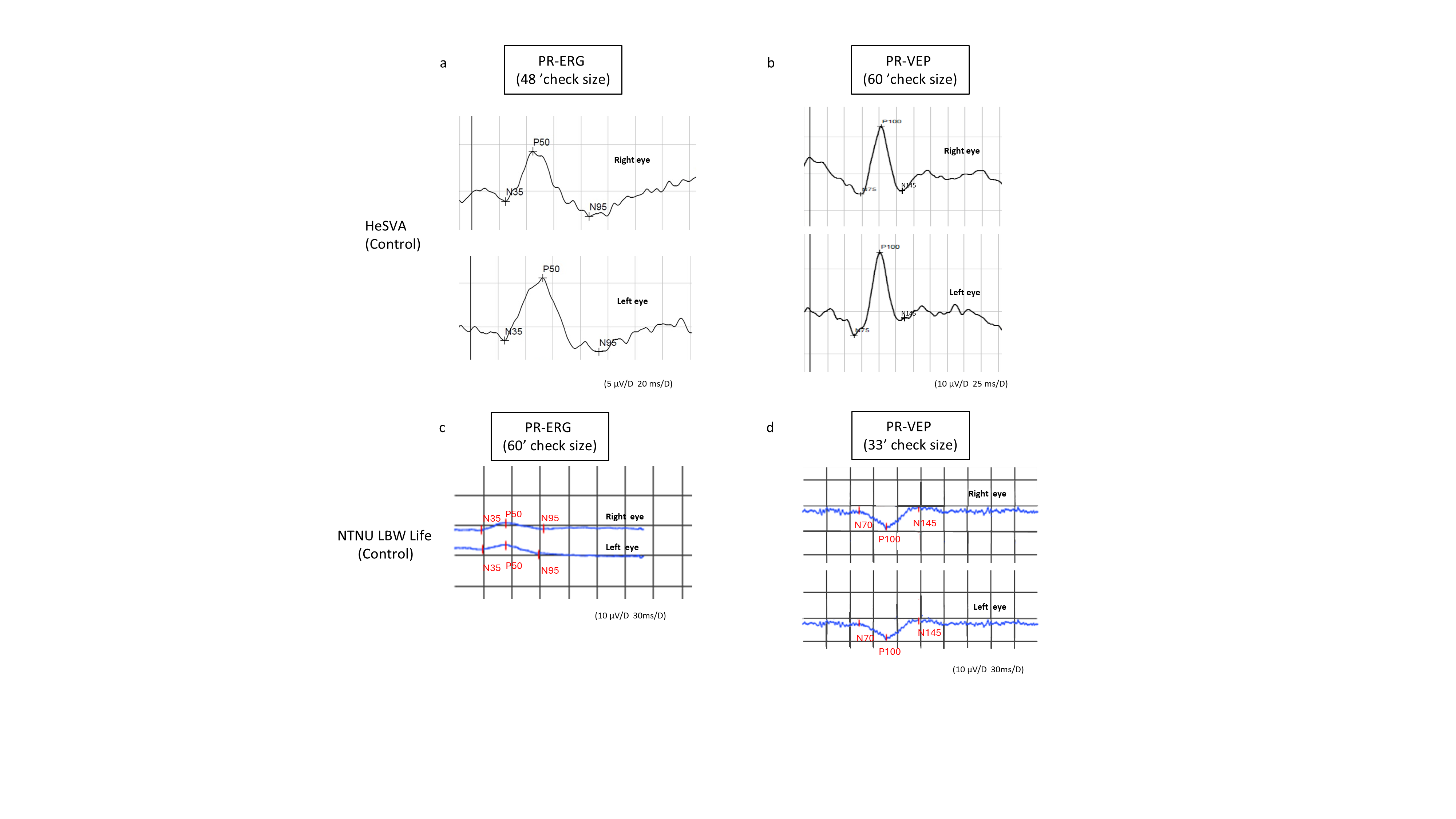

Supplement: Supplementary file 2 — Supplementary file2 (PNG 391 kb) Figure 2S. Examples of mean pattern reversal ERG (PR-ERG) and VEP (PR-VEP) responses from two representative control subjects, one in HeSVA (a, b) and one in NTNU LBW Life (c, d). Abbreviations: HeSVA Helsinki Study of Very Low Birth Weight Adults; NTNU LBW Life NTNU Low Birth Weight in a Lifetime Perspective study; PR-ERG pattern reversal electroretinogram; PR-VEP pattern reversal visual evoked potential; µV microvolt; ms milliseconds; D division. [file 10633_2025_10025_MOESM2_ESM.png]
